# MicroRNAs in Endometriosis: Insights into Inflammation and Progesterone Resistance

**DOI:** 10.3390/ijms241915001

**Published:** 2023-10-09

**Authors:** Jing-Xian Hon, Norhazlina Abdul Wahab, Abdul Kadir Abdul Karim, Norfilza Mohd Mokhtar, Mohd Helmy Mokhtar

**Affiliations:** 1 Department of Physiology, Faculty of Medicine, Universiti Kebangsaan Malaysia, Kuala Lumpur 56000, Malaysia; 2 GUT Research Group, Faculty of Medicine, Universiti Kebangsaan Malaysia, Kuala Lumpur 56000, Malaysia; 3 Department of Obstetrics & Gynecology, Faculty of Medicine, Universiti Kebangsaan Malaysia, Kuala Lumpur 56000, Malaysia

**Keywords:** endometriosis, inflammation, progesterone resistance, microRNA

## Abstract

Endometriosis, a non-malignant gynecological disorder influenced by estrogen, involves the growth of endometrial tissue outside the uterus. Its development includes processes such as inflammation, progesterone resistance, angiogenesis, and cell proliferation. Epigenetic factors, particularly the dysregulation of microRNAs (miRNAs), have emerged as key factors in these mechanisms in endometriosis. This review aims to unveil the intricate molecular processes that control inflammation, progesterone resistance, and miRNA functions in endometriosis. In addition, it provides a comprehensive overview of the current understanding regarding the involvement of miRNAs in the inflammatory aspects of this condition. This synthesis encompasses research investigating the molecular underpinnings of inflammation, along with the biogenesis and roles of miRNAs in endometriosis. Furthermore, it examines human studies and functional analyses to establish the intricate connection between miRNAs, inflammation, and progesterone resistance in the context of endometriosis. The results highlight the significant impact of dysregulated miRNAs on the inflammatory pathways and hormonal imbalances characteristic of endometriosis. Consequently, miRNAs hold promise as potential non-invasive biomarkers and targeted therapeutic agents aimed at addressing inflammation and enhancing the response to progesterone treatment in individuals with endometriosis.

## 1. Introduction

Endometriosis, a benign gynecological disease affecting approximately 10–15% of women of reproductive age, is characterized by the presence and growth of endometrial tissue outside the uterine cavity [1,2,3]. The disease manifests in various sites, with the ovaries being the most affected (67%), followed by the uterosacral ligaments (46%), ovarian fossa (32%), pouch of Douglas (30%), and bladder (21%) [4]. Endometriosis is classified into three subtypes: superficial endometriosis, deep infiltrating endometriosis (DIE), and ovarian endometrioma (also known as chocolate cysts) [5]. The leading theory of pathogenesis is retrograde menstruation, in which endometrial tissue flows back through the fallopian tubes and implants in the pelvic cavity, leading to chronic inflammation [3,6,7,8]. Coelomic metaplasia, proposed by Meyer, suggests that mesothelial cells differentiate into functional endometrium, which is an alternative theory for the development of endometriosis [6,7]. Other contributing factors may include immunological and epigenetic factors, such as microRNAs (miRNAs) as well as environmental and lifestyle factors, such as diet and exposure to dioxin and polychlorinated biphenyls (PCBs) [7]. A recent study has shown that the gut microbiota and microbiota-derived metabolites promote the growth of endometriotic lesions in mice, which could potentially be used as non-invasive biomarkers for endometriosis [9].

Endometriosis patients present with several common symptoms, including chronic pelvic pain, dysmenorrhea, dyspareunia, dysuria, dyschezia, and infertility [2,6,8]. The mechanisms underlying endometriosis include interdependent processes of endometrial proliferation, inflammatory pathways, and angiogenesis [8]. Inflammation in endometriosis is driven by elevated levels of macrophages and cytokines, including interleukins (IL-1β, IL-6, IL-8, IL-17), tumor necrosis factor α (TNFα), cyclooxygenase 2 (COX-2), and macrophage inhibitory factor (MIF) [8,10]. In addition, imbalances in sex hormones, specifically high levels of biologically active estradiol (E2) and low levels of progesterone receptors (PR), contribute to progesterone resistance and the dependence on estrogen in endometriosis [5,8,11]. Progesterone therapy utilizing synthetic compounds known as progestins is commonly used to treat endometriosis because of its antiproliferative and anti-inflammatory effects [12]. However, due to the phenomenon of progesterone resistance, the response to progestins varies from patient to patient, leading to unpredictable outcomes [13,14,15].

Epigenetic dysregulations, particularly miRNAs, are thought to contribute to the development of endometriosis. These small RNA molecules interact with target mRNAs and influence processes such as hypoxic injury, survival, proliferation, inflammation, remodeling, angiogenesis, and steroidogenesis, including progesterone resistance [13,16]. Consequently, miRNAs have the potential to be non-invasive biomarkers and targeted therapies for endometriosis [16,17]. Thus, more funding is needed for researchers to conduct more studies on the role of miRNAs in endometriosis so that the manipulation of miRNAs in our healthcare system would be possible. However, clinical management of infertility associated with endometriosis could be challenging due to its heterogeneity. The different phenotypes of patients make it difficult to make an accurate diagnosis and find a specific mechanism for this infertility. Therefore, the treatment of infertility should be personalized according to the clinical situation and degree of impairment [18]. Thus, the aim of this review is to provide insight into the role of miRNAs in inflammation and their contribution to the pathophysiology of endometriosis.

## 2. Dysregulation of Immune Mediators in Endometriosis

**Figure 1:**
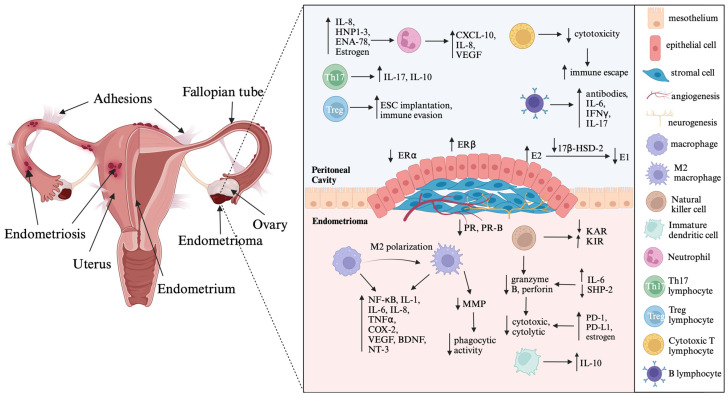
Illustration depicting the cascades of inflammatory processes intertwined with hormonal imbalances in endometriosis. Created with BioRender.com. Abbreviations: IL—interleukin; HNP1–3—human neutrophil peptides 1–3; ENA—epithelial neutrophil-activating peptide; VEGF—vascular endothelial growth factor; ESC—endometrial stromal cells; IFNγ—interferon γ; ER–estrogen receptor; E2—estradiol; E1—estrone; 17β-HSD-2—17β-hydroxysteroid dehydrogenase type 2; PR—progesterone receptor; KAR—killer activating receptor; KIR—killer inhibitory receptor; NF-κB—nuclear factor-κB; TNFα—tumor necrosis factor α; COX-2—cyclooxygenase 2; BDNF—brain-derived neurotrophic factor; NT-3—neurotrophin-3; MMP—matrix metalloproteinase; SHP-2—Src homology region 2-containing protein tyrosine phosphatase-2; PD-1—programmed cell death protein 1; PD-L1—programmed cell death ligand 1; The arrows indicate dysregulation of immune mediators produced by immune cells and steroid hormone imbalances in endometriotic lesion.

The pathophysiology of endometriosis includes inflammation and fibrosis, which contribute to common symptoms such as pelvic pain, dysuria, dyschezia, and infertility [5]. Endometriotic lesions have high levels of important inflammatory mediators such as cyclooxygenase-2 (COX-2), interleukins (IL-1β, IL-6, IL-8, IL-17), tumor necrosis factor-α (TNFα), prostaglandin E2 (PGE_2_), and estradiol (E2). These mediators interact with each other and exacerbate the inflammatory process in the tissue [5,8]. COX-2 expression is higher in the ectopic tissue of endometriosis patients than in the endometrium of women with and without this disease [5]. IL-1β stimulates COX-2 expression via the mitogen-activated protein kinase (MAPK) signaling pathway, further enhancing the inflammatory response in endometriosis [5,10,19]. In addition, upregulation of COX-2 increases PGE_2_ production, which in turn, increases aromatase (CYP19A1) activity and E2 synthesis [5,8]. This local inflammatory environment supports inflammation, angiogenesis, and survival of the endometriotic lesion through a positive feedback loop involving excessive PGE_2_ and E2 production [5]. IL-1β also stimulates angiogenesis and neurogenesis by promoting the production of vascular endothelial growth factor (VEGF), IL-6, nerve growth factor (NGF), and brain-derived neurotrophic factor (BDNF) [20,21]. VEGF, stimulated by E2, facilitates endothelial cell growth, migration, vasodilation, and increased vascular permeability [8,10,19]. Moreover, IL-1β enhances angiogenesis by upregulating the expression of angiogenic factors such as VEGF and IL-8 [22]. Women with endometriosis exhibit higher expression of VEGF, predominantly in peritoneal fluid macrophages, compared to healthy women [21]. IL-6 increases haptoglobin secretion and diminishes phagocytosis, which promotes survival and development of endometriotic lesions [20,21]. IL-17A induces the production of angiogenic factors (VEGF and IL-8), proinflammatory cytokines (IL-6 and IL-1β), and chemokines (G-CSF, CXCL12, CXCL1, and CX3CL1), promoting the growth of endometriotic lesions [20,23]. Additionally, IL-6 and TNFα facilitate adhesion of endometrial cells to the peritoneum, contributing to disease progression in endometriosis [20,21] (Figure 1).

The nuclear factor-κB (NF-κB) signaling pathway plays a crucial role in both cancer and endometriosis [5]. In endometriosis, iron overload stimulates activation of inhibitor of NF-κB kinase subunit beta (IKKβ) and leads to an increase in reactive oxygen species (ROS), activating the NF-κB pathway [5]. Iron overload also induces endometrial cell migration through activation of the NF-κB pathway [5,24]. Studies have shown that NF-κB expression increases in endometrial stromal cells (ESCs) and ectopic endometriotic lesions, which stimulate the production of proinflammatory cytokines, interleukins, TNFα, regulated on activation, normal T cell expressed and secreted (RANTES), and macrophage-migration inhibitory factor (MIF) [25]. Moreover, peritoneal macrophages in endometriosis patients show increased activation of the NF-κB pathway [26]. Activation of macrophages via the NF-κB signaling pathway leads to the release of interleukins, TNFα, COX-2, and VEGF, which further promotes inflammation in endometriosis [10,27]. The NF-κB pathway is involved in lipopolysaccharide (LPS)-induced inflammation in murine endometriotic lesions [5,28]. In addition, the NF-κB inhibitor disulfiram has shown promise in reversing the inflammatory process and preventing the growth of endometriotic tissue in animal models [5,29].

Oxidative stress may also play a role in inflammation and cell proliferation in endometriosis. It is characterized by elevated levels of ROS in cells, which induce the production of proinflammatory cytokines via NF-κB activation, promote angiogenesis, and contribute to extracellular matrix (ECM) degradation [30]. High levels of ROS have been detected in the peritoneal fluid of patients with endometriosis [31]. Previous studies have demonstrated that the administration of antioxidant enzymes can prevent adhesion formation in the peritoneal cavity of patients with endometriosis [32].

Following inflammation, the subsequent fibrotic process replaces active lesions with fibrotic tissue, leading to the formation of white lesions known as adhesions. These surviving lesions exhibit reduced inflammatory clearance and altered immune surveillance, contributing to the persistence of symptoms in endometriosis [5].

## 3. Dysregulation of Immune Cells in Endometriosis

Immune cell dysregulation plays a crucial role in the pathogenesis of endometriosis and contributes to chronic inflammation and disease progression. In this review, we will discuss the involvement of different types of immune cells in endometriosis, including neutrophils, macrophages, natural killer (NK) cells, and dendritic cells (DC) [20,21].

Neutrophils are key contributors to the initial progression of endometriosis. They are recruited to the sites of endometriotic lesions by IL-8; human neutrophil peptides 1, 2, and 3 (HNP1–3); and epithelial neutrophil-activating peptide (ENA-78). Estrogen affects the number of neutrophils and increases the expression of proinflammatory cytokines [20,21]. Neutrophils secrete CXCL-10, IL-8, and VEGF, which further exacerbates the inflammatory response in endometriosis [20].

Macrophages play a critical role in endometriosis, but their functions are impaired in the disease. Decreased expression of matrix metalloproteinases (MMPs) in macrophages leads to diminished phagocytic activity, which impedes the clearance of cellular debris [8,10]. Macrophages in endometriosis can be divided into M1 and M2 subtypes, with M1 macrophages promoting inflammation and M2 macrophages involved in immune regulation, fibrosis, immune tolerance, and angiogenesis. The balance between M1 and M2 macrophages shifts during disease progression, with the number of M1 macrophages decreasing and the number of M2 macrophages increasing. This suggests a transition from active inflammation to fibrosis in the advanced stages of endometriosis [33]. Macrophages also secrete prostaglandins, proinflammatory cytokines, and hydrolytic enzymes, contributing to the local inflammatory milieu. High levels of IL-10 in endometriosis play a significant role by decreasing the activity of CD4+ T-cells in the peritoneal fluid of affected patients [34]. Elevated IL-10 levels may contribute to polarization of M1 to M2 macrophages, as evidenced by an increase in CD163+ biomarkers expressed by M2 macrophages and a decrease in CD86+ biomarkers expressed by M1 macrophages [35]. Macrophages are not only present in ectopic endometriotic lesions but also in the eutopic endometrium and peritoneal fluid of patients with endometriosis [36,37]. In endometriosis, macrophages showed increased secretion of prostaglandins (PG), proinflammatory cytokines, and hydrolytic enzymes with increased levels of PG observed in peritoneal fluid [10,38]. Macrophage-induced oxidative stress may contribute to the development of chronic inflammation in endometriosis [20]. Greaves et al., showed that macrophages labeled with green fluorescent protein (GFP^+^) were present in endometriotic lesions after reciprocal transfers of shed endometrium between MacGreen and wild-type mice [39]. The interaction between macrophages and nerve fibers in endometriotic lesions may contribute to the manifestation of pain symptoms [20,21]. In response to estradiol, nerve fibers stimulate the secretion of colony-stimulating factor (CSF)-1 and C-C motif ligand (CCL)-2, which triggers macrophage migration [40]. Estradiol also stimulates macrophages to increase levels of brain-derived neurotrophic factor (BDNF) and neurotrophin (NT)-3, which promotes neurogenesis in endometriosis. Niclosamide has been shown to reduce macrophage-triggered inflammation via STAT3 and NF-κB signaling in human endometriotic stromal cells [41].

NK cells are crucial components of the innate immune system and play a role in endometriosis. In endometriosis, NK cells show reduced cytotoxic activity due to the absence of perforin and granzyme B in their lytic granules [8]. Additionally, the expression of killer activating receptors (KARs) is decreased, while killer inhibitory receptors (KIRs) are increased, particularly in advanced stages of the disease [20,21,42]. Elevated levels of IL-6 in the peritoneal fluid have been found to suppress Src homology region 2-containing protein tyrosine phosphatase-2 (SHP-2), resulting in decreased cytolytic activity of NK cells through downregulation of granzyme B and perforin [20,21]. In a study by Yang et al. (2017), co-cultured NK cells, macrophages, and ESCs from endometriosis patients demonstrated that the interaction between ESCs and macrophages reduced the cytotoxicity of NK cells by inducing the production of IL-10 and TGFβ [43]. Endometriotic lesions exhibit increased expression of the immune checkpoint molecules programmed cell death protein 1 (PD-1) and programmed cell death ligand 1 (PD-L1), which negatively affect NK cell function and contribute to immune abnormalities [20]. Estrogen has been shown to inhibit the cytotoxicity of NK cells by suppressing autophagy of ESCs, thereby promoting proliferation of endometriotic lesions [20].

Furthermore, the precise role of dendritic cells (DCs) in endometriosis remains unclear. However, immature DCs (imDCs) are thought to promote angiogenesis and ESC migration through secretion of IL-10 [44]. Further research is needed to unravel the precise involvement of DCs in the pathophysiology of endometriosis.

T and B lymphocytes of the adaptive immune system play an important role in the pathogenesis of endometriosis [20,21]. T helper 17 (Th17) cells, which produce IL-17 and IL-10, are increased in the peritoneal fluid of patients with endometriosis and promote angiogenesis and inflammation [45]. Regulatory T (Treg) cells contribute to the development of endometriotic lesions by facilitating the implantation of ESCs and promoting immune defense through local immunosuppression [20,21,46]. Cytotoxic T lymphocytes exhibit reduced cytotoxicity, which facilitates immune escape of endometriotic lesions. Hormonal changes have been observed to suppress activated subpopulations of T cells and impede NK cell cytotoxicity [20]. Osuga et al. (2016) reported increased levels of Th2 and Th17 cells in endometriotic tissue. IL-4 was found to stimulate the proliferation of ESCs, while IL-17A was shown to enhance the migration of neutrophils in endometriosis. Furthermore, the combination of IL-17A and TNFα stimulates the secretion of IL-8 and C-C motif chemokine ligand 20 (CCL-20) indicating the existence of interactions between the inflammatory response and Th17 cells [47].

B lymphocytes produce antibodies against the endometrium, contributing to the chronic inflammation and severity of the disease [20]. These antibodies can be detected in both the serum and peritoneal fluid and are associated with infertility and disease severity. In addition, B cells secrete cytokines, including IL-6, interferon γ (IFNγ), and IL-17, which also contribute to the inflammatory processes in endometriosis [20,21].

Thus, the dysregulation of immune cells in endometriosis contributes to chronic inflammation and disease progression. Neutrophils, macrophages, NK cells, DCs, and lymphocytes exhibit altered functions in endometriosis, resulting in impaired immune responses and the promotion of inflammation, angiogenesis, and immune evasion. Understanding the intricate interactions between immune cells and the pathophysiology of endometriosis will provide valuable insights for the development of targeted therapeutic approaches. Further research is warranted to unravel the complex immune dysregulation in endometriosis and its potential impact on clinical management.

## 4. Steroid Hormones Imbalances in Endometriosis

In endometrial tissue, an intriguing interplay between progesterone receptors (PRs) and estrogen receptors (ERs) has been unveiled. Notably, there is an imbalance with decreased PR activity compared to increased ER activity. The prevalent therapeutic approaches, predominantly based on progesterone and antiestrogen treatments, highlight the clinical significance of these imbalances.

The challenges posed by resistance to progestins, commonly referred to as progesterone resistance, represent a significant obstacle in the effective treatment of endometriosis [5]. The complex interplay between two PR isoforms, PR-A and PR-B, is gaining important as studies reveal an altered PR-A/PR-B ratio within endometriotic lesions [48,49,50,51,52,53]. The conspicuous absence of the PR-B isoform is emerging as a potential linchpin in the pathogenesis of endometriosis, with multiple implications for inflammation, cell proliferation, and complicated hormonal interactions [5,11] (Figure 1).

Another contributor to the intricate tapestry of endometriosis pathophysiology is the intricate web of ER signaling. Dysregulation of ER subtypes, particularly an elevation in ERβ combined with a concomitant decline in ERα expression, shifts the balance towards a proinflammatory environment [54,55,56,57,58,59]. Notably, ERβ plays a pivotal role by amplifying inflammatory processes through interactions with inflammasome components such as caspase 1 and NOD-like receptor protein 3 (NLRP3) and complex modulation of apoptotic pathways [8,55,60,61] as shown in Figure 1.

The core of these hormonal imbalances lies in the dysregulated conversion of estrogenic compounds. Altered expression of 17β-hydroxysteroid dehydrogenase type 2 (17β-HSD-2) coupled with compromised progesterone response leads to increased biologically active estradiol (E2) levels [62]. Moreover, increased aromatase activity contributes to local estrogen production, further fueling estrogen-driven cascades [5,8] (Figure 1).

The insights gained from the intricate interplay of the signaling pathways PR and ER and the enzymatic control of estrogen levels are promising for innovative therapeutic modalities. Targeted strategies aimed at restoring hormonal balance, such as selective ER modulators and agents that promote PR-B expression, are potential ways to alleviate the burdens associated with endometriosis.

## 5. MicroRNAs and Their Biogenesis

MicroRNAs (miRNAs) are a class of small non-coding RNAs about 22 nucleotides in length, originally discovered in *Caenorhabditis elegans*, where they play a pivotal role in regulating larval development [63,64]. Following transcription, miRNAs play a crucial role in modulating gene expression by binding to the target messenger RNA (mRNA), thereby inducing mRNA degradation or translational inhibition [64]. MiRNAs are present intracellularly as well as in extracellular bodily fluids with remarkable stability, such as plasma, breast milk, saliva, and cerebrospinal fluid [64,65,66]. They exert control over diverse biological processes including cell proliferation, differentiation, and apoptosis [63,67,68]. Dysregulated miRNA expression is seen in a spectrum of diseases ranging from heart disease to cancer, metabolic disorders, inflammatory conditions, and female gynecological disorders [64,67]. Female reproductive disorders encompassing cervical cancer, ovarian cancer, endometrial cancer, pre-eclampsia, recurrent miscarriage, and endometriosis exhibit distinct miRNA expression profiles [64,69]. Consequently, miRNAs have the potential to serve as clinical diagnostic biomarkers not only for cancer but also for several other diseases such as viral infections, neurodegenerative disorders, and diabetes [70,71,72]. To date, the miRNA database (version 22.1) catalogues 2812 human miRNA genes [73]. These genes can be either intergenic or within introns of other genes [74].

Biogenesis of miRNA begins with transcription of the miRNA gene by the RNA polymerase II or III, yielding primary miRNA (pri-miRNA) transcripts, which are then capped and polyadenylated. Subsequently, the pri-miRNA forms a complex with DiGeorge syndrome critical region 8 (DGCR8), which is cleaved by the enzyme Drosha (RNAse III) to yield precursor miRNA (pre-miRNA). Exportin 5 and RAN-GTP facilitate the export of the pre-miRNA from the nucleus to the cytoplasm. Further processing involves cleavage of pre-miRNA by another RNAse III enzyme, DICER, resulting in a double-stranded duplex. The duplex unwinds, with one miRNA strand undergoing degradation. The remaining mature miRNA strand associates with the RNA-induced silencing complex (RISC), composed of DICER, a TAR RNA-binding protein (TRBP), and Argonaute (Argo) protein [74,75]. This mature miRNA then binds to 3′-untranslated region (3′-UTR) of the target mRNA, leading to mRNA degradation or translational inhibition, depending on the degree of complementarity. Perfect binding leads to mRNA degradation and gene silencing, while limited binding leads to translational inhibition [74]. It is worth noting that a single miRNA can target multiple mRNAs through distinct cellular pathways, while a single mRNA can be targeted by multiple miRNAs [64,76]. Consequently, miRNA biogenesis is a multifaceted process in which one miRNA can regulate numerous mRNAs due to its concise sequence [77]. Recent findings also suggest miRNA-mediated regulation of gene expression at the 5′-untranslated region (5′-UTR) and the DNA coding sequence of the target mRNA [74].

## 6. MicroRNAs and Their Involvement in Inflammation within Endometriosis

Dysregulated expression of miRNAs has been shown to contribute to the inflammatory processes associated with endometriosis [16]. Notably, inhibition of miR-138 expression promotes inflammation by increasing levels of *TNF-α*, *IL-1β*, *IL-6*, and *IL-18* through activation of the *NF-κB* signaling pathway and *VEGF* in endometriosis [78]. Within this intricate web, proinflammatory cytokines such as *IL-1β*, *TNF-α*, and *transforming growth factor beta-1* (*TGF-β1*) suppress the *chicken ovalbumin upstream promoter-transcription factor II (COUP-TFII*) via miR-302a. This miRNA binds directly to the 3′UTR of *COUP-TFII* mRNA, leading to its degradation. This reduction in the expression of *COUP-TFII*, in turn, leads to an increase in *COX-2* expression in endometriotic lesions [79]. Noteworthy reports of circulating miRNAs have highlighted the decreased presence of the let-7 family, including let-7b-5p, both in endometriosis patients and in animal studies [80,81]. Reduced expression of *ERα*, *ERβ*, *aromatase*, *KRAS*, and *IL-6* has been demonstrated with the let-7b treatments. These genes highlight the multifaceted role of let-7b in endometriosis, which includes estrogen signaling, inflammation, and KRAS variants [82,83]. In patients with endometriosis, the upregulation of miRNA 125b-5p and downregulation of let 7b-5p have been associated with increased proinflammatory cytokines such as *TNF-α*, *IL-1β*, and *IL-6*. Transfection of macrophages with a miRNA 125b-5p mimic or let-7b-5p inhibitor has also been demonstrated to increase levels of these proinflammatory cytokines [84].

Distinctly, miR-33b has been found to be downregulated in endometriotic tissues. Notably, transfection of cultured endometrial cells with a miR-33b inhibitor resulted in increased endometrial proliferation as well as increased expression of *VEGF* and *MMP-9* mRNA. Interestingly, this was accompanied by a decrease in caspase-3 activity, a key player in apoptosis in endometriosis. Conversely, transfection with a miR-33b mimic resulted in decreased proliferation of *VEGF* and *MMP-9* mRNA with a concomitant increase in caspase-3 activity [85]. MiR-146b showed an increase in the peritoneal fluid of endometriosis patients, especially those in pain. In vitro studies using macrophages co-cultured with ESC showed increased miR-146b expression which is closely linked to the *NF-κB* pathway. Remarkably, miR-146b targets *IRF5* and effectively suppresses M1 macrophage activation, thereby reversing M1 polarization [86]. Moreover, the expression of miR-182 was downregulated and *RELA* upregulated in ESCs with increased inflammation and epithelial-mesenchymal transition (EMT), while the binding of miR-182 with *RELA* reduced inflammation, EMT, proliferation, migration, and invasion of ESCs via the *NF-κB* signaling pathway [87]. Schneider et al. (2013) found that the expression of miR-10b was downregulated and *SDC1* expression was upregulated, resulting in increased *IL-6* secretion, promoting inflammation in endometriosis [88].

A previous study on miRNA profile and cytokine content in peritoneal fluid of patients with endometriosis was conducted [89]. Nine miRNAs associated with inflammation, angiogenesis, proteolysis, or endometriosis (miR-29c-3p, miR-106b-3p, miR-130a-3p, miR-150-5p, miR-185-5p, miR-195-5p, miR-451a, miR-486-5p, and miR-1343-5p) were selected from the microarray data for validation. The validation results showed increased levels of miR-106b-3p, miR-451a, miR-486-5p, *IL-6*, *IL-8*, *urokinase plasminogen activator (uPA)*, and *tissue inhibitor of metalloproteinases type 1 (TIMP-1)* in the peritoneal fluid from patients with endometriosis. In the menstrual phase, the peritoneal fluid of patients with endometriosis had higher levels of miR-106b-3p, miR-130a-3p, miR-150-5p, miR-185-5p, miR-451a, miR-486-5p, *VEGF-A*, *IL-8*, *macrophage inflammatory protein 1β (MIP 1β)*, *uPA*, and *plasminogen activator inhibitor-1 (PAI-1)* compared to the proliferative and secretory phases. In addition, peritoneal fluid from infertile endometriosis patients showed higher miR-486-5p expression compared to infertile control patients. The expression of *VEGF-A, IL-8*, and *TIMP-1* was increased in the peritoneal fluid of infertile and fertile endometriosis patients compared to fertile patients without endometriosis [89].

Another study investigated whether induction of TNFα leads to dysregulation of miRNA expressions, which are related to the *NF-κB* signaling pathway and contribute to inflammation in endometriosis [90]. MiRNAs associated with inflammation in endometriosis (miR-125b-5p, miR-126-5p, miR-132-3p, miR-146a-5p, miR-15b-5p, miR-152-3p, miR-155-5p, miR-181a-5p, miR-196b-5p, miR199a-5p, miR-21-5p, miR-214-3p, miR-222a-3p, miR-23a-5p, miR-29b-3p, and miR-98-5p) were selected for analysis in ESCs from endometriosis patients and normal patients without endometriosis. The expressions of miR-126-5p, miR-132-3p, miR-15b-5p, miR-152-3p, miR-155-5p, miR-181a-5p, miR-196b-5p, miR199a-5p, miR-21-5p, miR-214-3p, miR-222a-3p, miR-23a-5p, miR-29b-3p, and miR-98-5p were significantly downregulated in ESCs from patients with endometriosis compared to patients without endometriosis. However, only miR-125b-5p showed a significant upregulation in ESCs from patients with endometriosis, while miR-146a-5p did not show significant differences between the two groups. In addition, in normal ESCs treated with TNFα, the expression of all selected miRNAs also decreased except for miR-146a-5p and miR-199a-5p, which were more highly expressed in normal ESCs treated with TNFα. TNFα also increased phosphorylations of the *PI3K*, *AKT*, *ERK*, and *NF-κB* signaling pathways. Circumin treatment has been shown to significantly increase the expression of selected miRNAs in ESCs from endometriosis patients, which also downregulates the expression of TNFα and the phosphorylations of the *AKT*, *ERK*, and *NF-κB* signaling pathways [90].

## 7. MicroRNAs and the Onset of Progesterone Resistance

Certain miRNAs are involved in promoting progesterone resistance in endometriosis by interacting with target mRNAs and ultimately causing mRNA degradation [13]. This process effectively suppresses genes associated with progesterone action. There is a clear link between endometriosis and fertility. In individuals without endometriosis, progesterone facilitates decidualization of the endometrium, a crucial step for a successful pregnancy. However, in women with endometriosis, the development of progesterone resistance hinders decidualization, promotes cell proliferation, and fuels inflammation in endometrial tissue [13,64].

Research involving baboons and women suffering from endometriosis has shown increased miR-29c expression with concomitant decreased *FKBP4* mRNA levels. Experiments conducted on human uterine fibroblast cells transfected with a miR-29c mimic showed decreased levels of both *FKBP4* and decidual markers [91]. Similarly, miR-135a and miR-135b were shown to regulate the expression of *homeobox A10 (HOXA10)* in women with endometriosis [92]. *HOXA10*, a gene regulated by progesterone, exhibits its highest expression during the implantation window. Thus, it is crucial for endometrial decidualization and embryo implantation [92,93].

Zhou et al. (2016) observed overexpression of miR-196a and upregulation of the *MEK/ERK* signaling in the eutopic endometrium of endometriosis patients. Transfection of ESCs with a miR-196a mimic led to increased *MEK/ERK* levels, accompanied by a concomitant decrease in the levels of *PR* and *PR-B*. Importantly, an impairment of decidualization was observed after transfection with the miR-196a mimic. In addition, inhibition of miR-196a resulted in a reversal of *MEK/ERK* signaling and an increase in the levels of *PR* and *PR-B* [94]. Additionally, miR-194-3p was identified as a factor contributing to decreased *PR* levels and impaired decidualization in the eutopic endometrium of endometriosis patients [95].

In the realm of progesterone resistance, miR-92a exerts its influence by suppressing *PTEN* mRNA expression, which further exacerbating the condition in endometriosis patients [96]. At the same time, increased expression of miR-297 decreases the expression of *PR* and impedes decidualization in the eutopic endometrial tissue of endometriosis patients [97]. The increased expression of miR-143-3p enhances the motility and invasiveness of endometriotic lesions, promoting disease progression due to an inadequate response to progesterone treatment [49]. Furthermore, miR-21-5p and *Hippo/yes-associated protein 1 (YAP1)* signaling pathway were upregulated, while the expression of *PR* was downregulated in endometriosis. Treatments with verteporfin (VP) and dienogest have been shown to downregulate miR-21-5p and *YAP1* expression, leading to upregulation of *PR* expression, thus improving progesterone resistance in endometriosis in humans and mouse models [98].

Other miRNAs have been validated as targets for *PR* leading to progesterone resistance in other diseases [49]. First, miR-126-3p directly targeted the 3′UTR of *PR* and downregulated the expression of *PR*, which also decreased the proliferation and expression of β-casein in mouse mammary epithelial cells [99]. MiR-129-2 also downregulated expression of *PR* when breast cancer cells were exposed to progesterone, while anti-miR-129-2 reversed this process and improved the response of patients to hormone treatment [100]. Three miRNAs (miR-513a-5p, miR-513b-5p, and miR-513c-5p) were significantly upregulated in breast cancer with miR-513a-5p strongly associated with breast cancer and inhibition of *PR* expression [101,102]. Toms et al. (2015) demonstrated that miR-378-3p reduced the expression of *PR* by directly binding to the 3′UTR of *PR* in ovarian granulosa cells, which in turn reduced the expression of *ADAMTS1*, *CTSL1*, and *PPARG* expressions in the ovary [103]. Another study demonstrated that miR-96 directly targeted the 3′UTR of *PR* in the endometrium of rhesus monkeys and humans, while miR-375 regulated the expression of *PR* only in rhesus monkeys, but not in humans and mice [104].

## 8. MicroRNAs Promoting Lesion and Progesterone Resistance in Endometriosis

Three studies profiling miRNA expression reported that miR-29c is increased in endometriotic lesions compared to eutopic endometrial tissue [67,105,106]. However, only Hawkins et al. (2011) performed another in vitro study on miR-29c using human endometrial stromal fibroblasts. The ECM genes (*COL7A1, UPK1B*, and *TFAP2C*), which are predicted targets of miR-29c were downregulated in cells transfected with miR-29c mimics. A luciferase reporter assay confirmed the binding of miR-29c to the 3′UTR of *COL7A1, COL21A1*, and *TFAP2C* ECM genes using human embryonic kidney (HEK) 293T cells [106]. Long and colleagues (2015) found that miR-29c expression was downregulated and c-Jun expression was upregulated in ectopic endometriotic lesions compared to eutopic endometrium in patients with and without endometriosis. The miR-29c mimic reduced cell proliferation and invasion in CRL-7566 endometriosis cell line, while induction of c-Jun reversed these processes. The miR-29c inhibitor, which increased cell proliferation and invasion, may contribute to the pathogenesis of endometriosis. Furthermore, the luciferase reporter assay showed that the 3′UTR of *c-Jun* is directly targeted by miR-29c [107]. The 3′UTR of *FKBP4*, which consists of the miR-29c-3p binding site, is largely conserved in vertebrates, while the 3′UTR of *PR*, which consists of the miR-29c-3p binding site is well conserved in mammals but poorly conserved in vertebrates [108]. Therefore, miR-29c-3p could target both *PR* and *FKBP4* in progesterone signaling. Most studies have found increased levels of miR-29c in endometriotic lesions, which could lead to progesterone resistance and lesion survival in endometriosis [49]. However, there are no studies identifying the direct binding of miR-29c to the 3′UTR of *PR*.

The expression of miR-143 was increased in ectopic endometriotic tissues compared to eutopic endometrial tissues [67,105]. Cosar et al. (2016) reported that miR-143-3p expression was upregulated in serum samples from women with endometriosis, while Papari et al. (2020) demonstrated that miR-143-3p expression was downregulated in plasma samples from women with endometriosis compared to control patients [109,110]. Yang et al. (2021) reported upregulation of miR-143-3p expression in ESCs compared to normal ESCs. However, functional studies showed conflicting results, with overexpressed miR-143-3p reducing ESC proliferation and invasion, while inhibition of miR-143-3p enhanced ESC proliferation and invasion. The authors concluded that miR-143-3p suppresses the progression of endometriosis, which may be a novel therapy for this disease [111]. Finally, Li and colleagues (2022) demonstrated that miR-143-3p expression was overexpressed in ectopic endometriotic tissues compared to eutopic and normal endometrium. Inhibition of miR-143-3p attenuated EMT, invasion, and migration of ESCs. MiR-143-3p was confirmed to directly bind to *vasohibin 1 (VASH1)* via activation of *TGF-β* signaling, promoting EMT, cell migration, and invasion in endometriosis [112]. These findings suggest that dysregulation of miR-143-3p may lead to progesterone resistance and increased progression of endometriotic lesions in endometriosis patients [49]. The studies discussed above are listed in Table 1.

## 9. Conclusions

In conclusion, this comprehensive review sheds light on the intricate interplay between miRNAs and inflammation, as well as their pivotal role in triggering progesterone resistance in the context of endometriosis. The aberrant expression of miRNAs has emerged as a significant contributor to the inflammatory processes associated with endometriosis, perpetuating a cycle of chronic inflammation, cell proliferation, and perturbed decidualization. Neutrophils, macrophages, NK cells, DCs, and lymphocytes exhibit altered functions in endometriosis, resulting in impaired immune responses and the promotion of inflammation, angiogenesis, and immune evasion. The nuanced mechanisms through which miRNAs modulate key inflammatory mediators, such as TNF-α, IL-1β, and IL-6, highlight their potential as influential regulators in the intricate network of molecular events underlying this condition.

Progesterone resistance, a hallmark of endometriosis, is closely intertwined with dysregulated miRNA expression, resulting in the disruption of crucial progesterone-driven processes such as decidualization. The orchestrated actions of specific miRNAs, such as miR-29c, miR-135a, miR-135b, and miR-196a, contribute to the intricate modulation of genes pivotal for hormonal responsiveness and tissue remodeling.

The insights gleaned from this review promise to inform future diagnostic and therapeutic strategies. The identification of miRNAs as key players in endometriosis-associated inflammation and progesterone resistance, suggests their potential as non-invasive biomarkers for early detection and monitoring of the disease. Moreover, the delineation of miRNA-mediated pathways offers a fertile ground for the development of targeted interventions aimed at ameliorating inflammation, restoring progesterone sensitivity, and ultimately improving patient outcomes.

As the landscape of miRNA research continues to evolve and the complex interactions within the endometriosis microenvironment are unraveled, new opportunities for innovative therapeutic approaches will emerge. Harnessing the potential of miRNAs as diagnostic tools and therapeutic agents could usher in a new era in the treatment of endometriosis, giving sufferers hope for a better quality of life and improved reproductive outcomes.

## Figures and Tables

**Table 1:** Studies of dysregulated miRNAs involved in inflammation, progesterone resistance, and lesion development in endometriosis.

Dysregulated miRNA	Targeted Gene or Pathway	Function	Sample Type	Reference
Downregulated: miR-138	Increased *TNF-α, IL-1β, IL-6, IL-18, NF-κB*, and *VEGF*	Inflammation and apoptosis	Uterine endothelial and THP-1 cells	Zhang et al. (2019) [78]
Upregulated: miR-302a	Decreased *COUP-TFII*, Increased COX-2	Inflammation	ESC	Lin et al. (2014) [79]
Upregulated: Let-7b	Decreased *ERα, ERβ, aromatase*, *KRAS* and *IL-6*.	Estrogen signaling, inflammation and KRAS	Endometriotic lesions	Sahin et al. (2018) [82]
Upregulated: miR-125b-5p Downregulated: Let 7b-5p	Increased *TNF-α, IL-1β, IL-6* and *IL-8*	Inflammation	Sera and human U937 macrophage cell line	Nematian et al. (2018) [84]
Downregulated: miR-33b	Increased *VEGF* and *MMP-9*, reduced caspase-3	Proliferation and apoptosis	Endometriotic tissues and cultured endometrial cells	Yang et al. (2017) [85]
Upregulated: miR-146b	Reduced *IRF5*	Inflammation	Peritoneal fluid and macrophages co-cultured with ESCs	Zhang et al. (2019) [86]
Downregulated: miR-182	Increased *RELA* and *NF-κB* pathway	Inflammation, EMT, proliferation, migration, and invasion.	ESCs	Wu et al. (2021) [87]
Downregulated: miR-10b	Increased *SDC1*	Inflammation and invasion	12Z endometriotic cell line and primary eutopic ESCs	Schneider et al. (2013) [88]
Upregulated: miR-106b-3p, miR-130a-3p, miR-150-5p, miR-185-5p, miR -451a, miR -486-5p	Increased *IL-6*, *IL-8*, *uPA*, *TIMP-1*, *VEGF-A*, *MIP 1β* and *PAI-1*	Inflammation, angiogenesis, and proteolysis	Peritoneal fluid	Marí-Alexandre et al. (2018) [89]
Downregulated: miR-126-5p, miR-132-3p, miR-15b-5p, miR-152-3p, miR-155-5p, miR-181a-5p, miR-196b-5p, miR199a-5p, miR-21-5p, miR-214-3p, miR-222a-3p, miR-23a-5p, miR-29b-3p, and miR-98-5p	Increased phosphorylation of *PI3K*, *AKT*, *ERK*, and *NF-κB*	Inflammation	ESCs	Banerjee et al. 2023 [90]
Upregulated: miR-29c	Reduced *FKBP4*	Progesterone resistance	Eutopic ESCs, HUF cells	Joshi et al. (2017) [91]
Upregulated: miR-135a and miR-135b	Reduced *HOXA10*	Progesterone resistance	Eutopic endometrium, ESCs, MCF-7 cells	Petracco et al. (2011) [92]
Upregulated: miR-196a	Increased *MEK/ERK*, reduced *PR* and *PR-B*	Progesterone resistance	Eutopic endometrium, ESCs	Zhou et al. (2016) [94]
Upregulated: miR-194-3p	Reduced *PR* and *PR-B*	Progesterone resistance	Eutopic endometrium, ESCs	Pei et al. (2018) [95]
Upregulated: miR-92a	Reduced *PTEN*	Progesterone resistance	Endometriotic tissue and ESCs	Li et al. (2020) [96]
Upregulated: miR-297	Reduced *PR*	Progesterone resistance	ESCs	Liu et al. (2023) [97]
Upregulated: miR-21-5p	Increased *YAP1* and reduced *PR*	Progesterone resistance	Endometriotic tissues, ESCs and serum	Lin et al. (2023) [98]
Upregulated: miR-29c	Reduced *COL7A1*, *UPK1B*, and *TFAP2C*	Lesion development and progesterone resistance	human endometrial stromal fibroblasts, HEK 293T cells	Hawkins et al. (2011) [106]
Downregulated: miR-29c	Increased *c-Jun*	Proliferation, invasion, and apoptosis	Eutopic endometrium, ectopic endometriotic lesion and CRL-7566 endometriosis cell line	Long et al. (2015) [107]
Upregulated: miR-143-3p	Reduced *autophagy-related 2B (ATG2B)*	Proliferation and invasion	ESCs	Yang et al. (2021) [111]
Upregulated: miR-143-3p	Reduced *VASH1*	EMT, migration and invasion	ESCs	Li et al. (2022) [112]
